# Differentiating atypical parkinsonian syndromes—a way forward?

**DOI:** 10.1002/brb3.341

**Published:** 2015-05-18

**Authors:** Ronald F Pfeiffer

**Affiliations:** Department of Neurology, University of Tennessee Health Science CenterMemphis, Tennessee

Any neurologist—even a movement disorders neurologist, if he or she is honest, will tell you that it can be maddeningly difficult to differentiate between Parkinson's disease (PD) and the atypical parkinsonian syndromes (APS) such as multiple system atrophy (MSA), progressive supranuclear palsy (PSP), and corticobasal degeneration (CBD), especially during their initial clinical stages. Although the cynic might propose that it does not make much of a difference, since the treatment measures employed are essentially identical, the reality is that accurate diagnosis does, indeed, make a difference to patients and their family members. The likelihood for a meaningful response to medication is markedly less for individuals with one of the APS (even though some individuals with MSA may achieve, at least initially, a noticeable response) and the expectations, prognosis and life expectancy for individuals with one of the APS are significantly bleaker than for individuals with PD. Patients and their family members want and are entitled to know this because it can make a tremendous difference in the counseling they receive and the planning they may make.

Accurate diagnosis also is important from a research standpoint. It has been documented that the initial diagnosis of PD is often incorrect. In a recent report comparing clinical diagnosis with neuropathological confirmation, the accuracy rate of general neurologists in diagnosing PD was only 75% (Joutsa et al. 2014) and in another recent report utilizing data from the Arizona Study of Aging and Neurodegenerative Disorders, the diagnostic accuracy of a clinical diagnosis of PD in untreated or poorly treatment-responsive individuals was only 26%, rising to 53% in individuals with suspected early PD responsive to medication and to a greater extent than 85% in individuals with a more long-standing diagnosis responsive to medication (Adler et al. 2014). Diagnostic accuracy in the APS is also notoriously suboptimal (Litvan et al. 1997a,b). Although an accuracy rate of 90% is achieved when the diagnosis is made by neurologists specializing in movement disorders utilizing specific diagnostic criteria (Hughes et al. 2001), much room for improvement still exists.

This diagnostic inaccuracy, especially early in the disease course, also has tremendous implications from the standpoint of clinical trials. Much research attention currently is focused on the search for agents that might modify disease progression, which would be most effective if employed early in the course of the disease, even before the appearance of motor dysfunction if possible, since by the time the motor features of PD become evident, approximately 50% of dopaminergic neurons have already been lost (Kordower et al. 2013). It is possible, in fact, that the error rate inherent in the purely clinical diagnosis of PD already has led to the failure to identify effective disease modifying agents because of the inclusion in clinical trials of subjects who later turned out to have one of the APS or other disorders.

It is amidst this quandary that an impressive array of approaches toward complementing and improving the accuracy of clinical diagnosis of PD and APS has undergone investigation. Approaches as varied as cerebrospinal fluid markers (Hall et al. 2012; Herbert et al. 2014; Magdalinou et al. 2015), smell testing (Driver-Dunckley et al. 2014), breath analysis (Nakhleh et al. 2015), voice analysis (Hariharan et al. 2014), transcranial ultrasonography (Fernandes Rde and Berg 2015), and others have been studied. However, it is neuroimaging in a variety of permutations that has received the most attention. Radiotracer imaging procedures, such as dopaminergic imaging with both PET and SPECT, and cardiac imaging with MIBG, already have found some usefulness in clinical practice. Advanced magnetic resonance imaging techniques, particularly those focusing on atrophy patterns, are now receiving increasing attention. Magnetic resonance volumetry is performed in a manual or semi-automated fashion that is operator dependent and time consuming; in contrast, voxel-based morphometry is a fully automated approach that is rater-independent and does not require prior identification of regions of interest (Holtbernd and Eidelberg 2014).

Yu and colleagues have taken the innovative and important step of submitting information from 39 published voxel-based morphometry articles, encompassing a total of 782 patients with either PD or one of the APS, to whole brain meta-analysis utilizing the modified Anatomic Likelihood Estimation method (Yu et al. 2015) and have identified characteristic patterns of gray matter atrophy, unique to each disorder, to be evident for each of the APS (MSA, PSP, CBD). This information will serve as a vital comparison point and guide for future studies.

However, hurdles remain. Although distinctive patterns of gray matter involvement have been identified for the APS with voxel-based morphometry, overlap is present and interpretation cannot currently be performed on individual patients. It also is unclear, as discussed by Yu and colleagues, how effective this method may be in identifying changes in early disease, since atrophy may be a later-occurring phenomenon. Perhaps even more problematic is the fact that in the vast majority of studies utilizing voxel-based morphometry—and in the vast majority of studies evaluating all types of potential biomarkers—the control against which the new techniques or procedures are compared is the clinical diagnosis; a standard which we know is inadequate because of errors in diagnosis, as described above. The “gold standard” of neuropathological confirmation of diagnosis is rarely employed and, thus, the best the various procedures can hope to be is equivalent to an already imperfect standard. Prospective, longitudinal studies that ultimately have neuropathological confirmation of diagnosis are sorely needed to jump this hurdle, but will be very challenging to complete.

## References

[b1] Adler CH, Beach TG, Hentz JG, Shill HA, Caviness JN, Driver-Dunckley E (2014). Low clinical diagnostic accuracy of early vs. advanced Parkinson disease: clinicopathologic study. Neurology.

[b2] Driver-Dunckley E, Adler CH, Hentz JG, Dugger BN, Shill HA, Caviness JC (2014). Olfactory dysfunction in incidental Lewy body disease and Parkinson's disease. Parkinsonism Relat. Disord.

[b3] Fernandes Rde C, Berg D (2015). Parenchymal imaging in movement disorders. Front. Neurol. Neurosci.

[b4] Hall S, Öhrfelt A, Constantinescu R, Andreasson U, Surova Y, Bostrom F (2012). Accuracy of a panel of 5 cerebrospinal fluid biomarkers in the differential diagnosis of patients with dementia and/or parkinsonian disorders. Arch. Neurol.

[b5] Hariharan M, Polat K, Sindhu R (2014). A new hybrid intelligent system for accurate detection of Parkinson's disease. Comput. Methods Programs Biomed.

[b6] Herbert MK, Eeftens JM, Aerts MB, Esselink RA, Bloem BR, Kuiperij HB (2014). CSF levels of DJ-1 and tau distinguish MSA patients from PD patients and controls. Parkinsonism Relat. Disord.

[b7] Holtbernd F, Eidelberg D (2014). The utility of neuroimaging in the differential diagnosis of parkinsonian syndromes. Semin. Neurol.

[b8] Hughes AJ, Daniel SE, Lees AJ (2001). Improved accuracy of clinical diagnosis of Lewy body Parkinson's disease. Neurology.

[b9] Joutsa J, Gardberg M, Röyttä M, Kaasinen V (2014). Diagnostic accuracy of parkinsonism syndromes by general neurologists. Parkinsonism Relat. Disord.

[b10] Kordower H, Olanow CW, Dodiya HB, Chu Y, Beach TG, Adler CH (2013). Disease duration and the integrity of the nigrostriatal system in Parkinson's disease. Brain.

[b11] Litvan I, Goetz CG, Jankovic J, Wenning GK, Booth V, Bartko JJ (1997a). What is the accuracy of the clinical diagnosis of multiple system atrophy? A clinicopathologic study. Arch. Neurol.

[b12] Litvan I, Agid Y, Goetz C, Jankovic J, Wenning GK, Brandel JP (1997b). Accuracy of the clinical diagnosis of corticobasal degeneration: a clinicopathologic study. Neurology.

[b13] Magdalinou NK, Paterson RW, Schott JM, Fox NC, Mummery C, Blennow K (2015). A panel of nine cerebrospinal fluid biomarkers may identify patients with atypical parkinsonian syndromes. J. Neurol. Neurosurg. Psychiatry.

[b14] Nakhleh MK, Badarny S, Winer R, Jeries R, Finberg J, Haick H (2015). Distinguishing idiopathic Parkinson's disease from other parkinsonian syndromes by breath test. Parkinsonism Relat. Disord.

[b15] Yu F, Barron DS, Tantiwongkosi B, Fox P (2015). Patterns of gray matter atrophy in atypical parkinsonism syndromes: a VBM meta-analysis. Brain and Behavior.

